# 371. Estimating SARS-CoV-2 Seroprevalence from Spent Blood Samples, January–March 2021

**DOI:** 10.1093/ofid/ofab466.572

**Published:** 2021-12-04

**Authors:** Daniel Graciaa, Hans Verkerke, Jeannette Guarner, Ana Maria Moldoveanu, Narayana Cheedarla, Connie Arthur, Andrew Neish, Sara Auld, Angie Campbell, John Roback, Neel Gandhi, Sarita Shah

**Affiliations:** 1 Emory University School of Medicine, Atlanta, Georgia; 2 Rollins School of Public Health, Atlanta, Georgia; 3 Emory University, Atlanta, Georgia

## Abstract

**Background:**

Measuring SARS-CoV-2 antibody prevalence in spent samples at serial time points can determine seropositivity in a diverse pool of individuals to inform understanding of trends as vaccinations are implemented.

**Methods:**

Blood samples collected for clinical testing and then discarded ("spent samples") were obtained from the clinical laboratory of a medical center in Atlanta. A convenience sample of spent samples from both inpatients (medical/surgical floors, intensive care, obstetrics) and outpatients (clinics and ambulatory surgery) were collected one day per week from January-March 2021. Samples were matched to clinical data from the electronic medical record. In-house single dilution serological assays for SARS-CoV-2 receptor binding domain (RBD) and nucleocapsid (N) antibodies were developed and validated using pre-pandemic and PCR-confirmed COVID-19 patient serum and plasma samples (Figure 1). ELISA optical density (OD) cutoffs for seroconversion were chosen using receiver operating characteristic analysis with areas under the curve for all four assays greater than 0.95 after 14 days post symptom onset. IgG profiles were defined as natural infection (RBD and N positive) or vaccinated (RBD positive, N negative).

Figure 1. Nucleocapsid serology assay validation

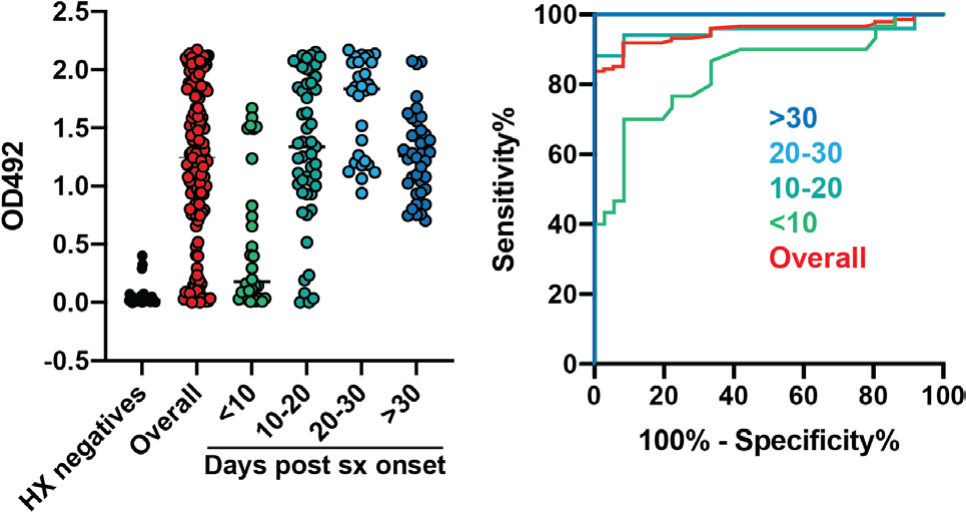

Single dilution serological assays for SARS-CoV-2 nucleocapsid antibodies were validated using pre-pandemic and PCR-confirmed COVID-19 patient serum and plasma samples. ELISA optical density (OD) cutoffs for seroconversion were chosen using receiver operating characteristic (ROC) analysis with areas under the curve (AUC) for all four assays greater than 0.95 after 14 days post symptom onset.

**Results:**

A total of 2406 samples were collected from 2132 unique patients. Median age was 58 years (IQR 40-70), with 766 (36%) ≥ 65 years. The majority were female (1173, 55%), and 1341 (63%) were Black. Median Elixhauser comorbidity index was 5 (IQR 2-9). 210 (9.9%) patients ever had SARS-CoV-2 detected by PCR, and 191 (9.0%) received a COVID-19 vaccine within the health system. Nearly half (1186/2406, 49.3%) of samples were collected from inpatient units, 586 (24.4%) from outpatient labs, 403 (16.8%) from the emergency department, and 231 (9.6%) from infusion centers. Overall, 17.0% had the IgG natural infection profile, while 16.2% had a vaccination profile. Prevalence estimates for IgG due to natural infection ranged from 24.0% in week 2 to 9.7% in week 5, and for IgG due to vaccine from 4.4% in week 2 to 32.0% in week 6 (Table, Figure 2).

Table. SARS-CoV-2 antibody seropositivity by week of sample collection for spent routine blood chemistry samples.

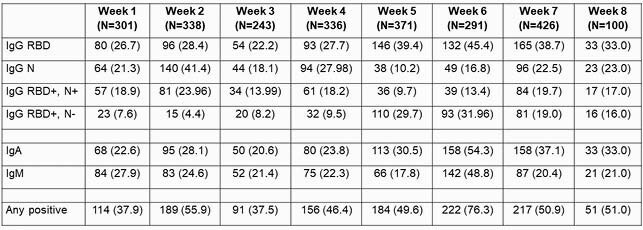

RBD = receptor binding domain. N = nucleocapsid. Seropositivity defined by enzyme-linked immunoassay (ELISA) optical density cutoffs selected using receiver operating characteristic analysis with areas under the curve (AUC) for all four assays greater than 0.95 after 14 days post symptom onset. IgG defined as positive if both RBD and N seropositive.

Figure 2. RBD and Nucleocapsid seropositivity to differentiate natural infection vs. vaccination by week of sample collection.

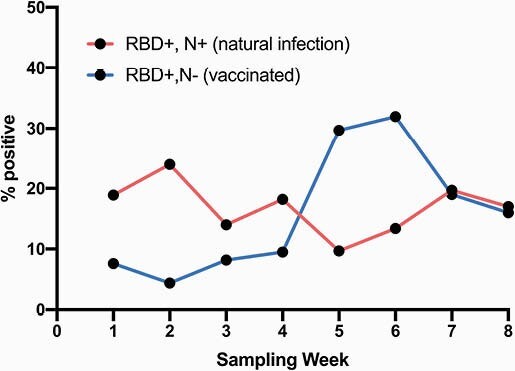

RBD = receptor binding domain. N = nucleocapsid. Seropositivity defined by enzyme-linked immunoassay (ELISA) optical density cutoffs selected using receiver operating characteristic analysis with areas under the curve (AUC) for all four assays greater than 0.95 after 14 days post symptom onset.

**Conclusion:**

Estimated SARS-CoV-2 IgG seroprevalence among patients at a medical center from January-March 2021 was 17% by natural infection, and 16% by vaccination. Weekly trends likely reflect community spread and vaccine uptake.

**Disclosures:**

**Daniel Graciaa, MD, MPH, MSc**, **Critica, Inc** (Consultant)

